# Supplementary figures and images for: A resource for the Drosophila antennal lobe provided by the connectome of glomerulus VA1v
Source: eLife. 2018 Nov 1;7:e37550. doi: 10.7554/eLife.37550 (PMC6234030; doi:10.7554/eLife.37550)

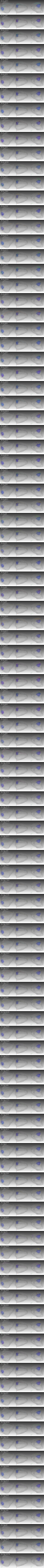

Supplement: Figure 4—source data 1. [file elife-37550-fig4-data1.pdf]

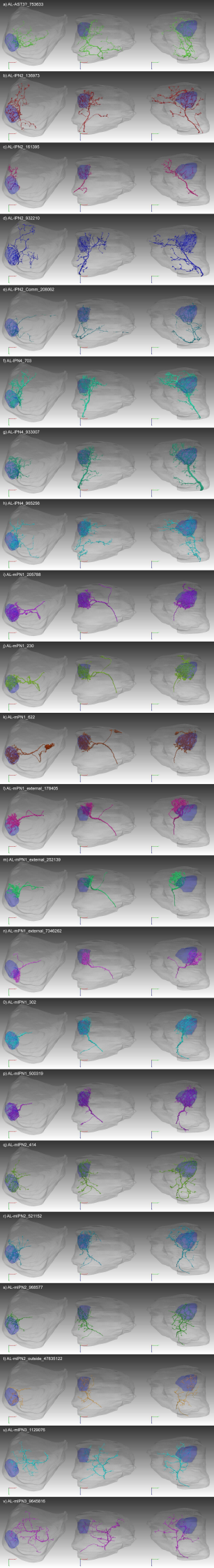

Supplement: Figure 4—source data 2. [file elife-37550-fig4-data2.pdf]

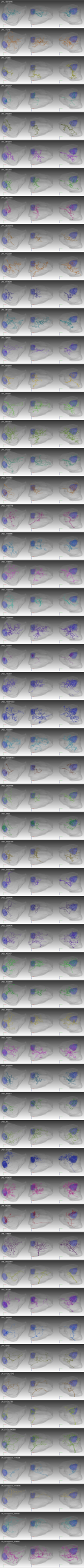

Supplement: Figure 4—source data 3. [file elife-37550-fig4-data3.pdf]

Orphan\_218396

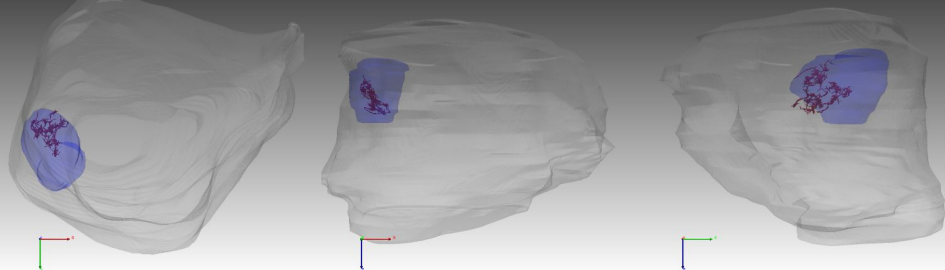

Orphan\_293930118

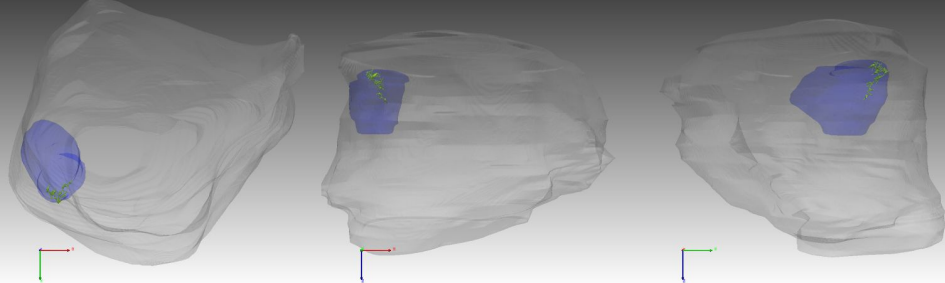

Orphan\_293931142

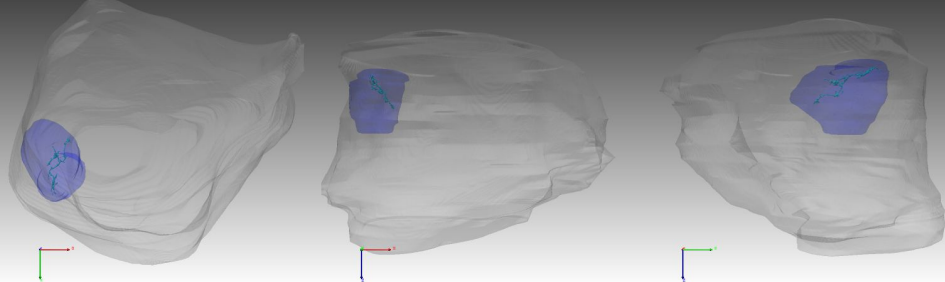

Orphan\_293933009

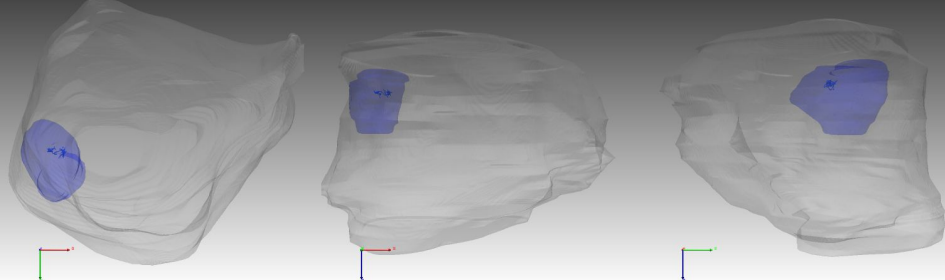

Orphan\_293933750

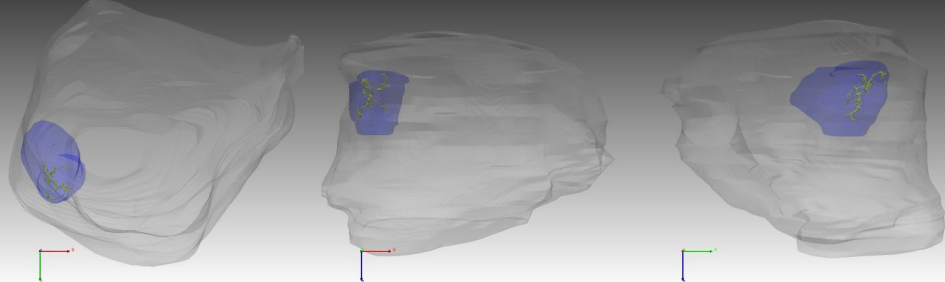

Orphan\_607253

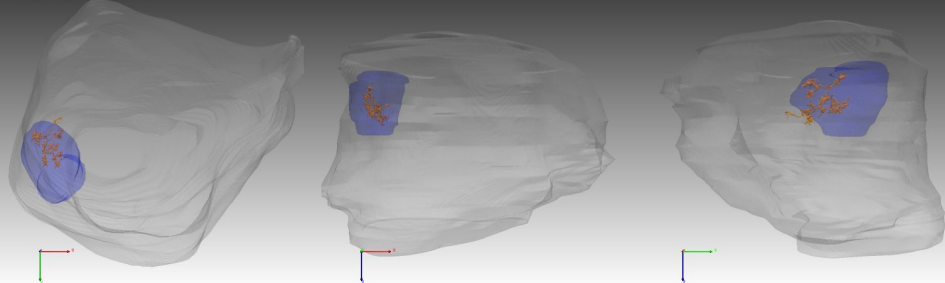

Unknown\_5161627

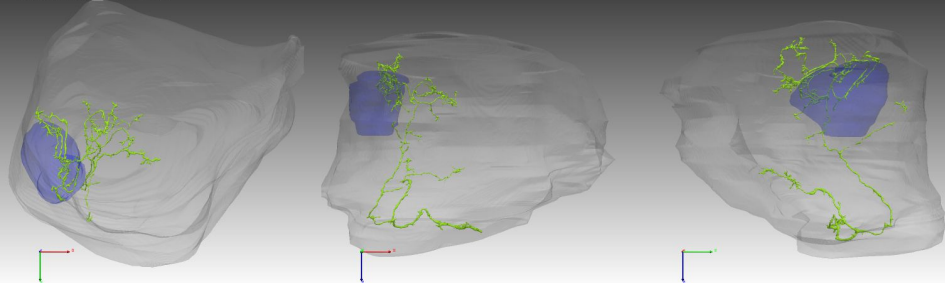

Supplement: Figure 4—source data 4. [file elife-37550-fig4-data4.pdf]
